# Supplementary material for: An invertible, invariant crystal representation for inverse design of solid-state materials using generative deep learning
Source: Nat Commun. 2023 Nov 2;14:7027. doi: 10.1038/s41467-023-42870-7 (PMC10622439; doi:10.1038/s41467-023-42870-7)
Supplement: Supplementary file 1 — Supplementary Information [file 41467_2023_42870_MOESM1_ESM.pdf]

Supplementary Information

An invertible, invariant crystal representation for inverse design of solid-state materials using generative deep learning

Xiao et al.

Supplementary Note 1. StructureMatcher algorithm

The StructureMatcher algorithm in Pymatgen[1] v.2022.11.7 first reduces the input crystal structures to their primitive cells and rescales them to equivalent volumes. The algorithm then searches for a valid affine mapping between the two cells, within predefined fractional length and angle tolerances. Finally, the maximum root-mean-square displacement between aligned structures normalized by the average free length per atom is computed. If below the site tolerance, the algorithm classifies the structures as similar based on the optimal lattice transformation found via permutation search.

Supplementary Note 2. Analysis of unsuccessful structure reconstruction by SLI2Cry

We analyzed four representative cases where SLI2Cry was unable to reconstruct original crystal structures (Supplementary Fig. 1). (a) For $TbSm_3$ (mp-1187379), the rescaled and ZL^*-optimized structure (2, 3) matches the original structure, but the M3GNet IAP optimization on ZL^*-optimized structure (3) encountered an “Exception encountered when calling layer spherical_bessel_with_harmonics” error. (b) For Cu_2O_3 (mp-755040), atomic collisions in the barycentric embedding led to a problematic rescaled structure (2), causing reconstruction failure. (c) $CdPb_2(ClO)_2$ (mp-1077904) exhibited underestimated bond lengths of ZL^*-optimized structure (3), affecting the reconstruction with M3GNet IAP. (d) For $Sm(HO)_3$ (mp-625409), the EconNN algorithm overestimated the coordination of certain hydrogen atoms, resulting in a poor ZL^*-optimized structure (3) and subsequent reconstruction failure. In summary, further improving the accuracy and robustness of modified GFN-FF in step (II) could enhance SLI2Cry's reconstruction performance.

Supplementary Note 3. Reconstruction performance of SLI2Cry for the filtered QMOF-21-40 dataset

QMOF-21-40 contains 928 MOFs with 21-40 atoms per unit cell from the Quantum MOF database[2]. After excluding MOFs containing atoms with atomic numbers beyond 86 and those with low-dimensional structural motifs, the filtered QMOF-21-40 dataset consists of 339 MOFs. Only 339 MOFs remained in the filtered QMOF-21-40 dataset, primarily owing to a large percentage of MOFs in the database contains low-dimensional components, as identified by the EconNN algorithm.

Supplementary Table 1 presents the reconstruction performance of SLI2Cry for the filtered QMOF-21-40 dataset. The match rates of 6.19% under loose criteria and 2.95% under strict criteria indicate that the current iteration of SLI2Cry faces challenges for reconstructing MOFs from SLICES strings. This can be primarily attributed to two factors: (1) The barycentric embedding from graph theory, used in SLI2Cry's step (I), might not provide suitable initial guesses for the organic components of MOFs. (2) The rotational degrees of freedom inherent to the organic linkers in MOFs hamper structural matching. While not presently invertible for MOFs, SLICES can still capture and store the chemical connectivity of MOFs.

Supplementary Note 4. Sampling speed of SLICES-based inverse design scheme

A workstation with dual Intel Xeon E5-2699v4 CPU (2x22 cores, 2.2 GHz) and a NVIDIA RTX 2080 Ti GPU was employed to run the inverse design scheme. The training of RNN models took ~ 14 hours, while sampling 10 million SLICES strings took ~6 hours. The reconstruction of approximately 3.4 million crystals from SLICES took under 6 days. Additionally, the screening process for identifying promising candidates took around 4 days. In total, 14 potentially synthetically accessible direct narrow-gap semiconductors with unique compositions and structures were inversely designed in less than 11 days on this workstation.

Supplementary Figure 1

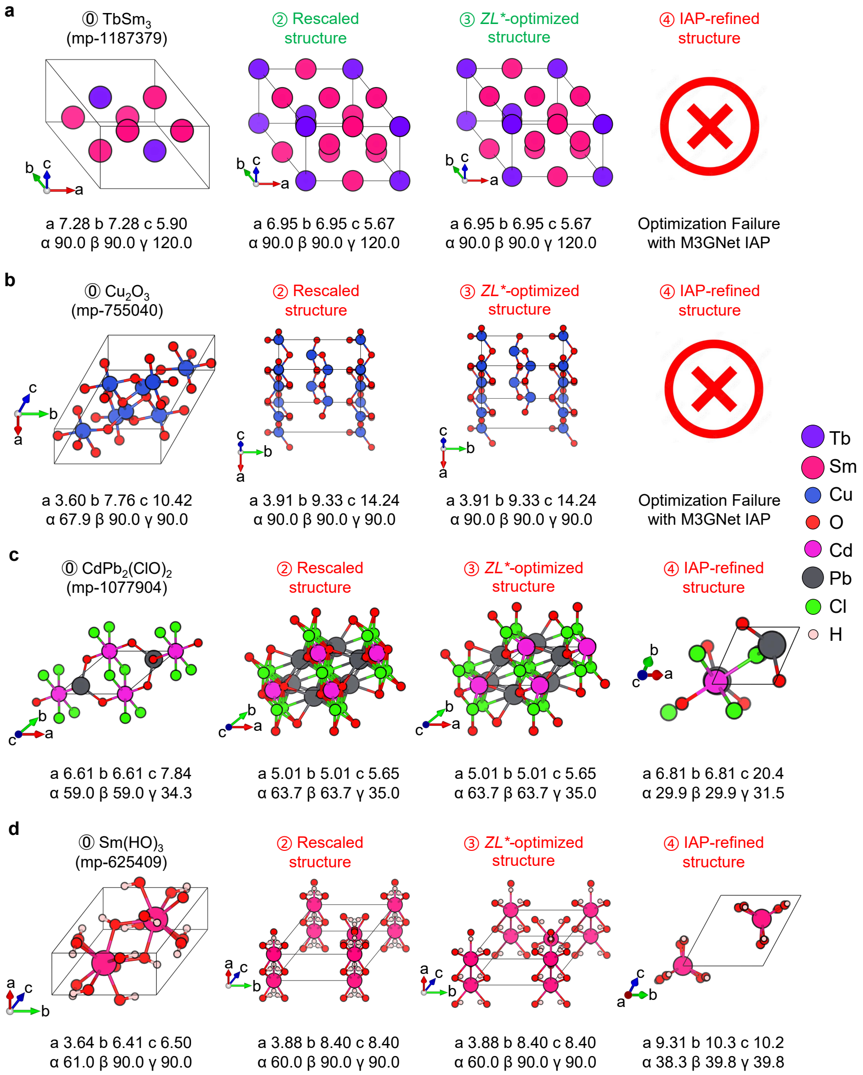

Supplementary Fig. 1 | Analysis of four failure cases of SLI2Cry for crystal structure reconstruction. The original (0), rescaled (2), ZL^*-optimized (3), and IAP-refined (4) structures of $TbSm_3$ (**a**), Cu_2O_3 (**b**), $CdPb_2(ClO)_2$ (**c**), $Sm(HO)_3$ (**d**). The lattice parameters are provided for each structure. Red error marks in the figure represent failed structural refinements using M3GNet IAP. The numbering scheme for structures in this figure is the same with that of Fig. 2 in the main text to ensure structural correspondence. Structure (1), the barycentric embedding, is not depicted here. The original structures are marked in black. Structures that match the original ones are marked in green, while those failing to match the original ones are marked in red.

Supplementary Figure 2

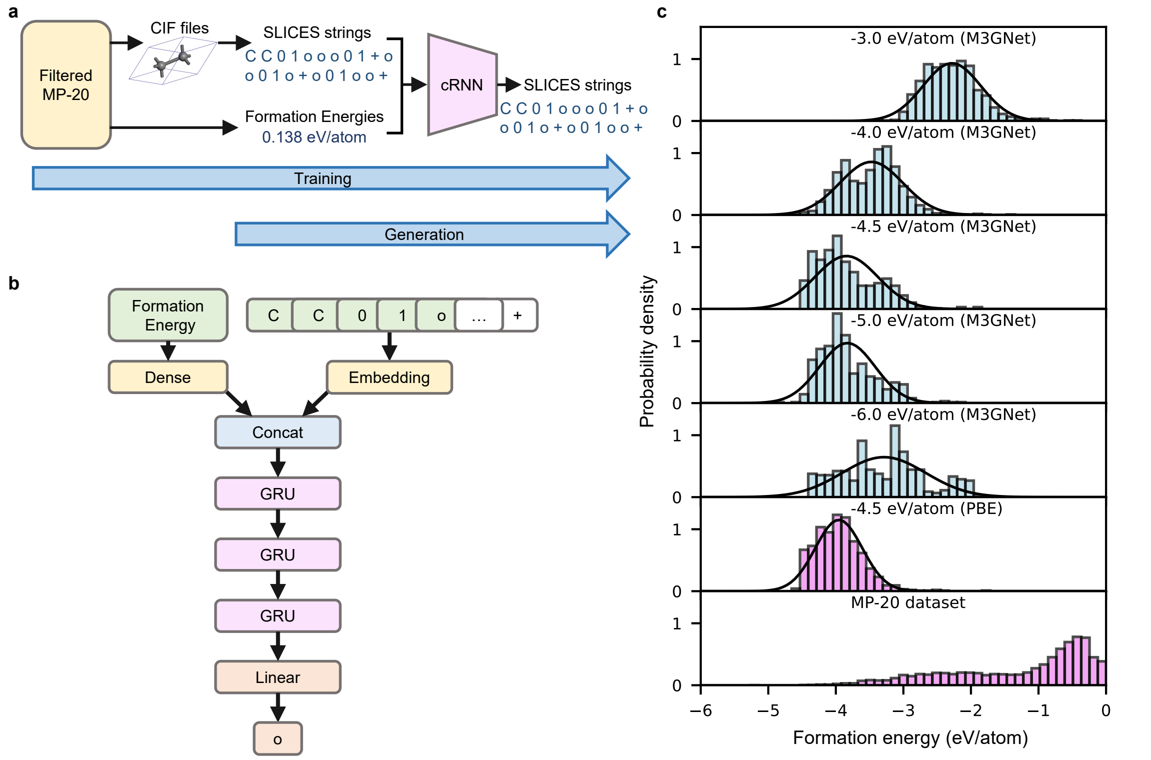

Supplementary Fig. 2 | Conditional RNN model for controlled generation of crystals with desired formation energy. a, Pipeline for training and controlled generation using the conditional RNN model. **b**, The conditional RNN model architecture. **c**, Distribution of formation energy of generated crystals under various user-specified targets ([-3.0, -4.0, -4.5, -5.0, -6.0] eV/atom), compared with the formation energy distribution of the MP-20 dataset. Normal distribution curves are fitted and included for the top six histograms. For the top five histograms, the formation energies were predicted using the M3GNet model. For M3GNet-predicted formation energies, the minimal mean value was obtained with a target of -4.5 eV/atom. For the second histogram from the bottom, the formation energies were calculated using PBE functional. Source data are provided as a Source Data file.

Supplementary Table 1 | Reconstruction performance of SLI2Cry on the filtered MP-21-40 dataset (23,560 crystals) and the filtered QMOF-21-40 dataset (339 MOFs)

Setting	Match rate (%)	
	Filtered MP-21-40	Filtered QMOF-21-40
Strict	83.73	2.95
Loose	87.88	6.19

Supplementary Table 2 | Parameters used in the models

**Model**	**Key parameters**	**Tasks trained for**	**Notes**
General RNN	Vocabulary size = 96, Embedding dimension = 128, GRU units = 512	Generating crystals as SLICES	Trained on Materials Project crystals with $N_{atom} \in [1,\ 10]$ and $E_{form} < 0$ for 10 epochs (30,085 SLICES; Augmented dataset: 764,546 SLICES)
Specialized RNN	Vocabulary size = 96, Embedding dimension = 128, GRU units = 512	Generating crystals with direct narrow-gap as SLICES	Trained on direct bandgap semiconductors in Materials Project with $E_g^{PBE} \in [0.1,\ 0.55]$, $N_{atom} \in [1,\ 10]$ and $E_{form} < 0$ for 8 epochs (364 SLICES; Augmented dataset: 11,373 SLICES)
Unconditional RNN	Vocabulary size = 106, Embedding dimension = 128, GRU units = 512	Generating crystals as SLICES for evaluating structural validity and compositional validity	Trained on the filtered MP-20 for 10 epochs (40,330 SLICES; Augmented dataset: 2,009,115 SLICES)
Conditional RNN	Vocabulary size = 106, Embedding dimension = 128, Dense layer dimension = 64, GRU units = 512	Generating crystals with desired formation energy as SLICES for evaluating success rate	Trained on the filtered MP-20 for 10 epochs (40,330 SLICES with E_{form}; Augmented dataset: 2,009,115 SLICES with E_{form})

Supplementary References

1. Ong, S. P. *et al.* Python Materials Genomics (pymatgen): A robust, open-source python library for materials analysis. *Comput. Mater. Sci.* **68**, 314–319 (2013).
2. Rosen, A. S. *et al.* Machine learning the quantum-chemical properties of metal–organic frameworks for accelerated materials discovery. *Matter* **4**, 1578–1597 (2021).
